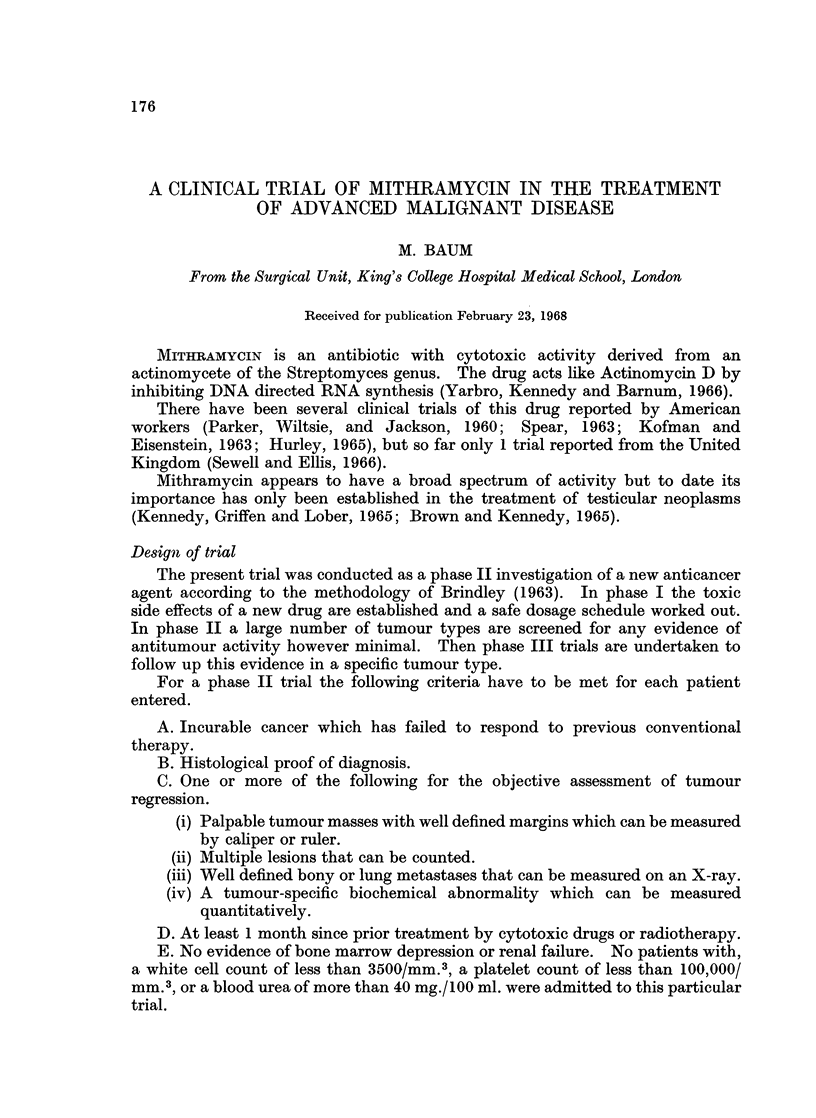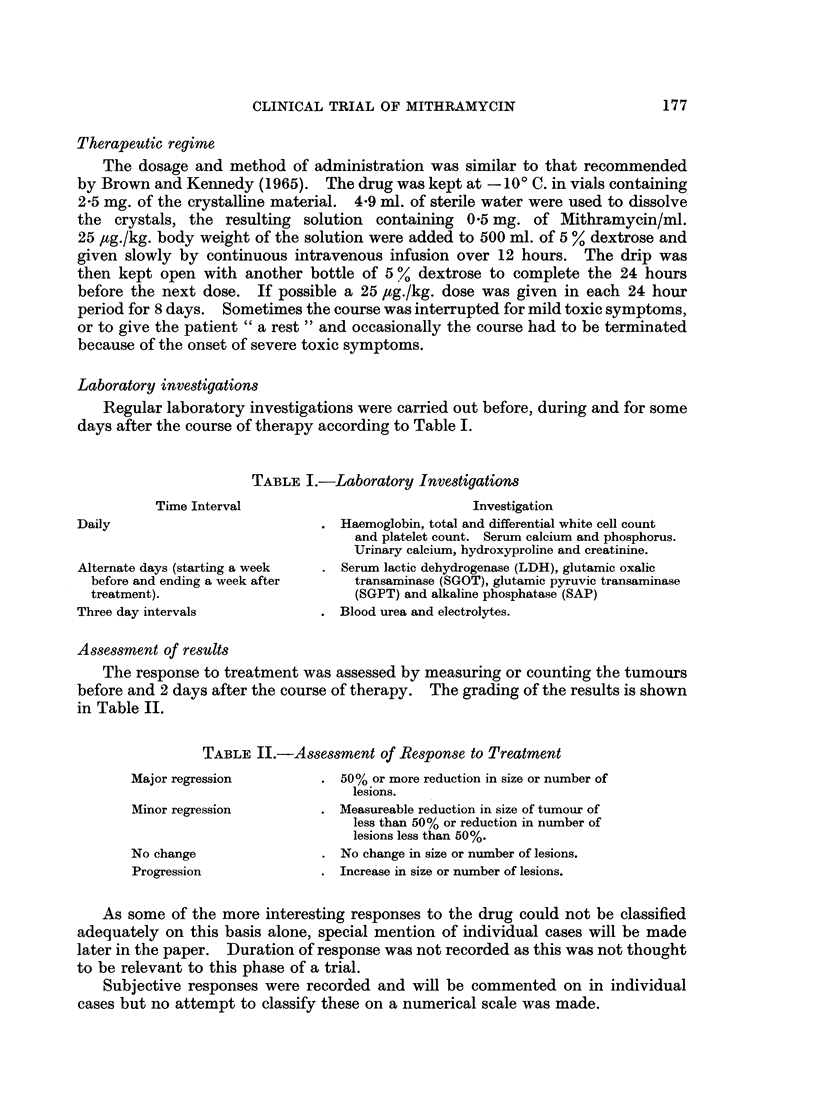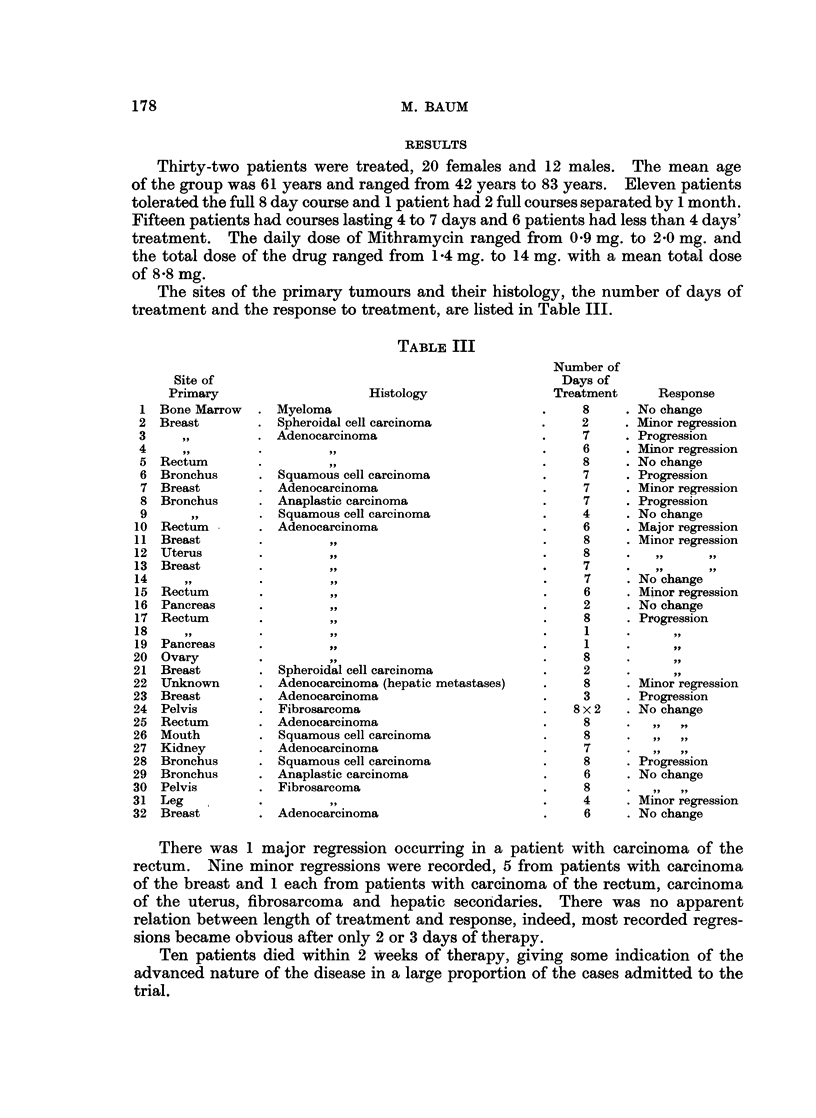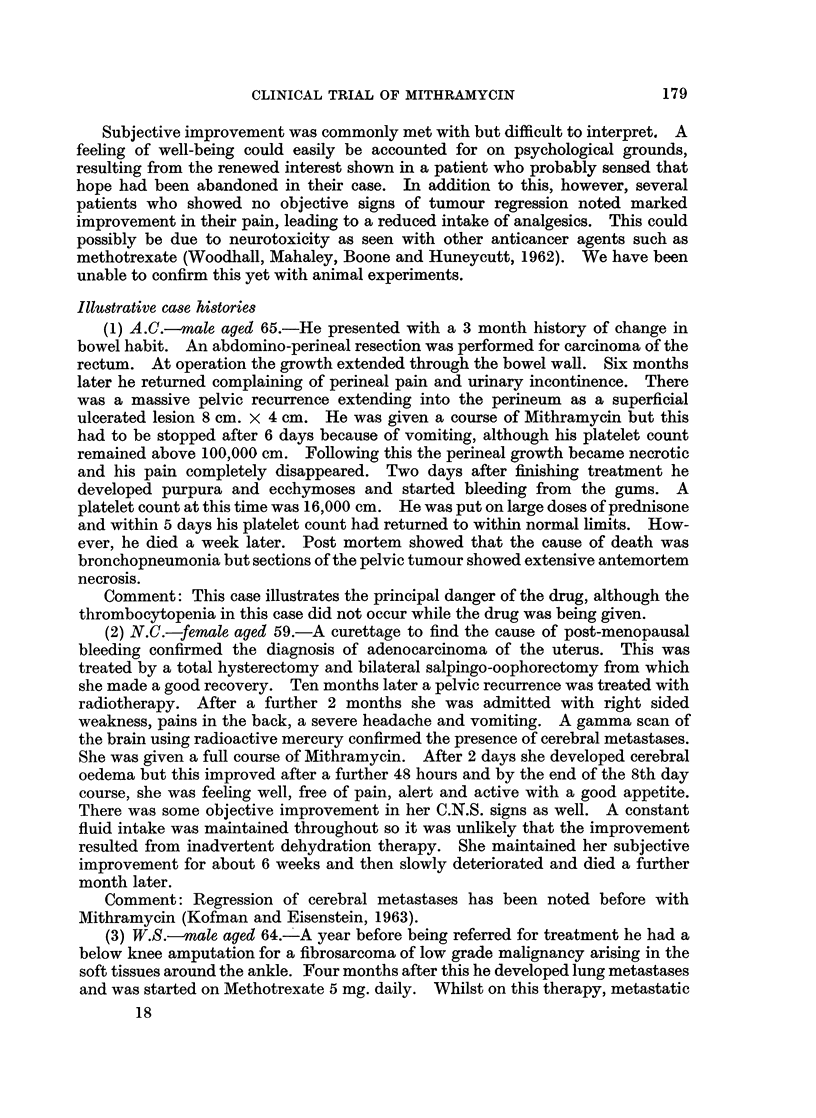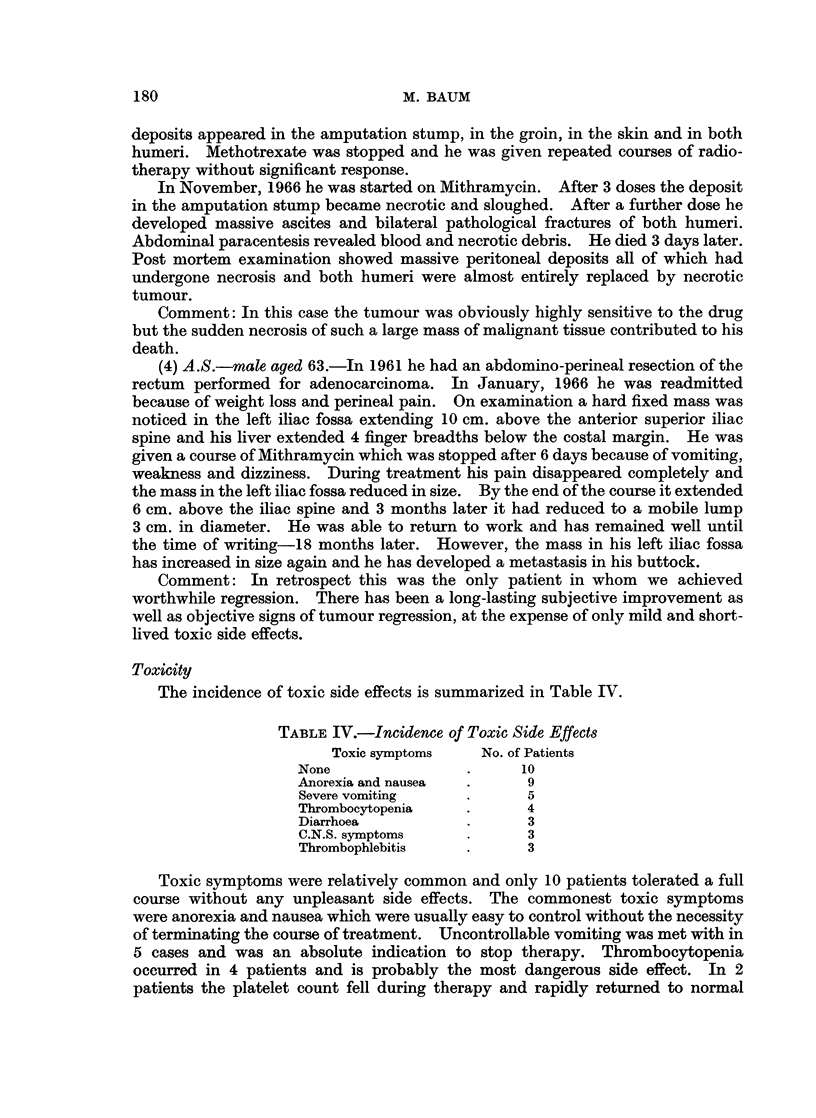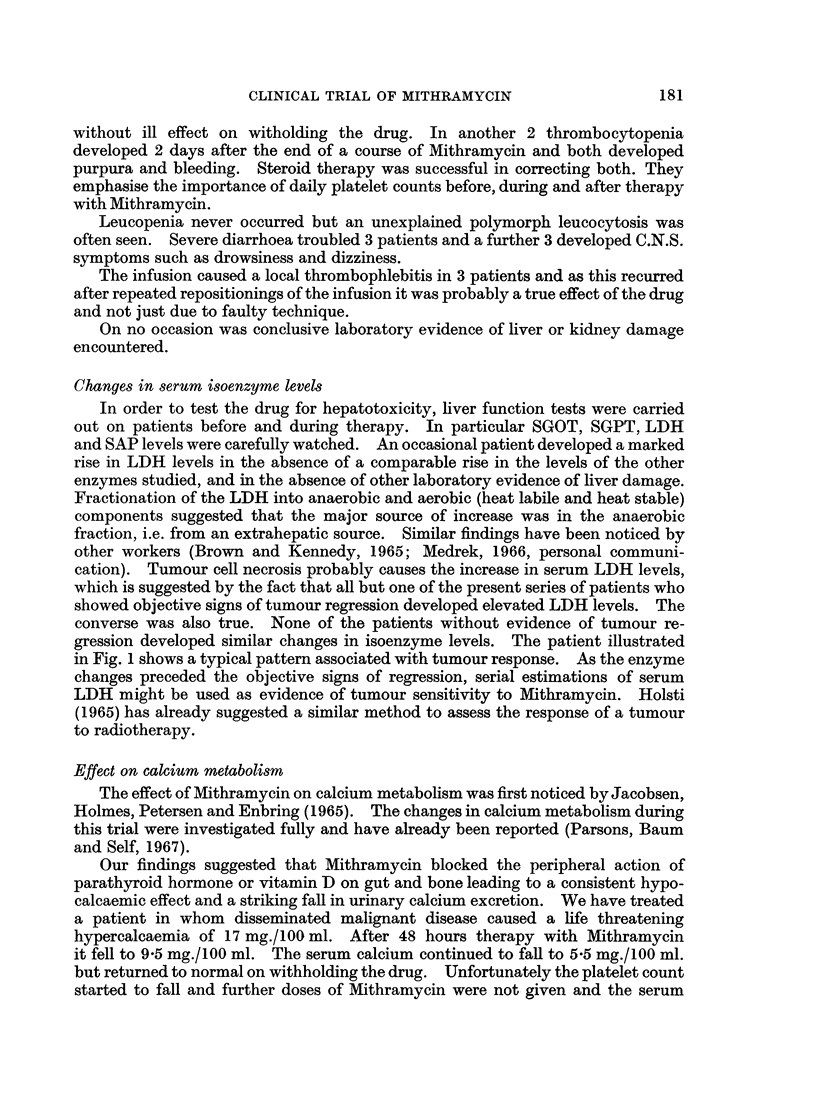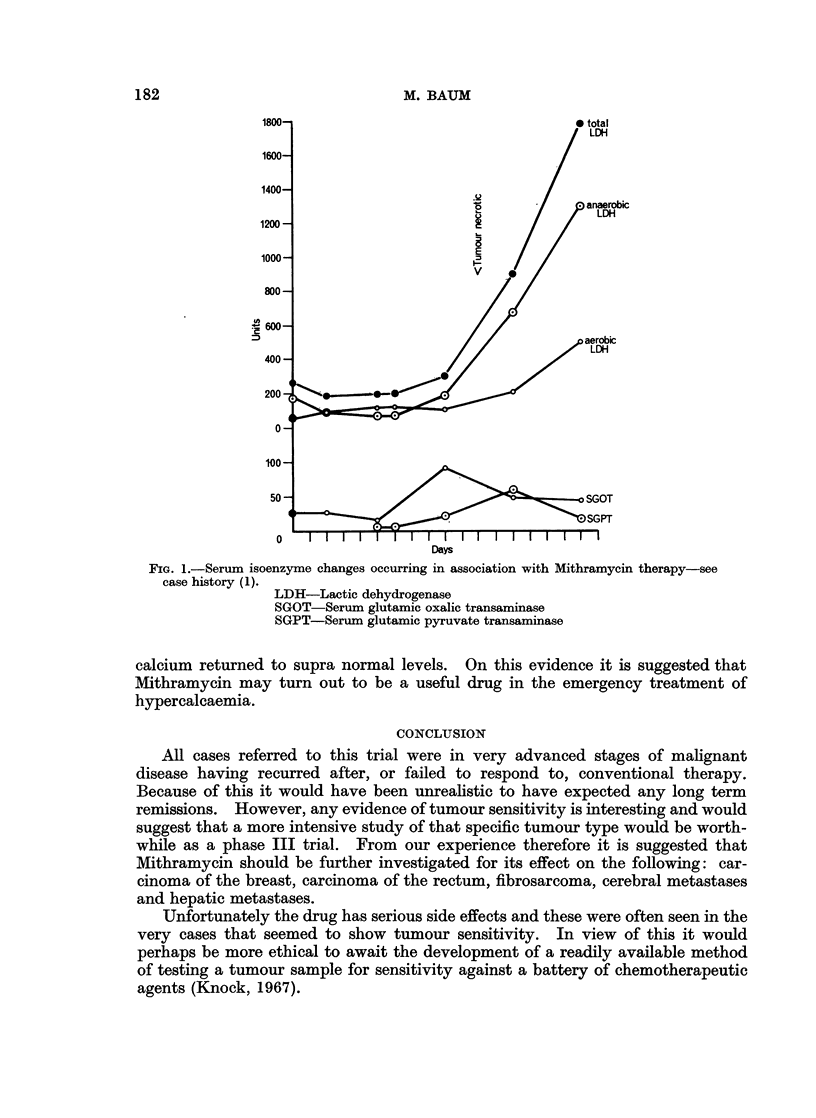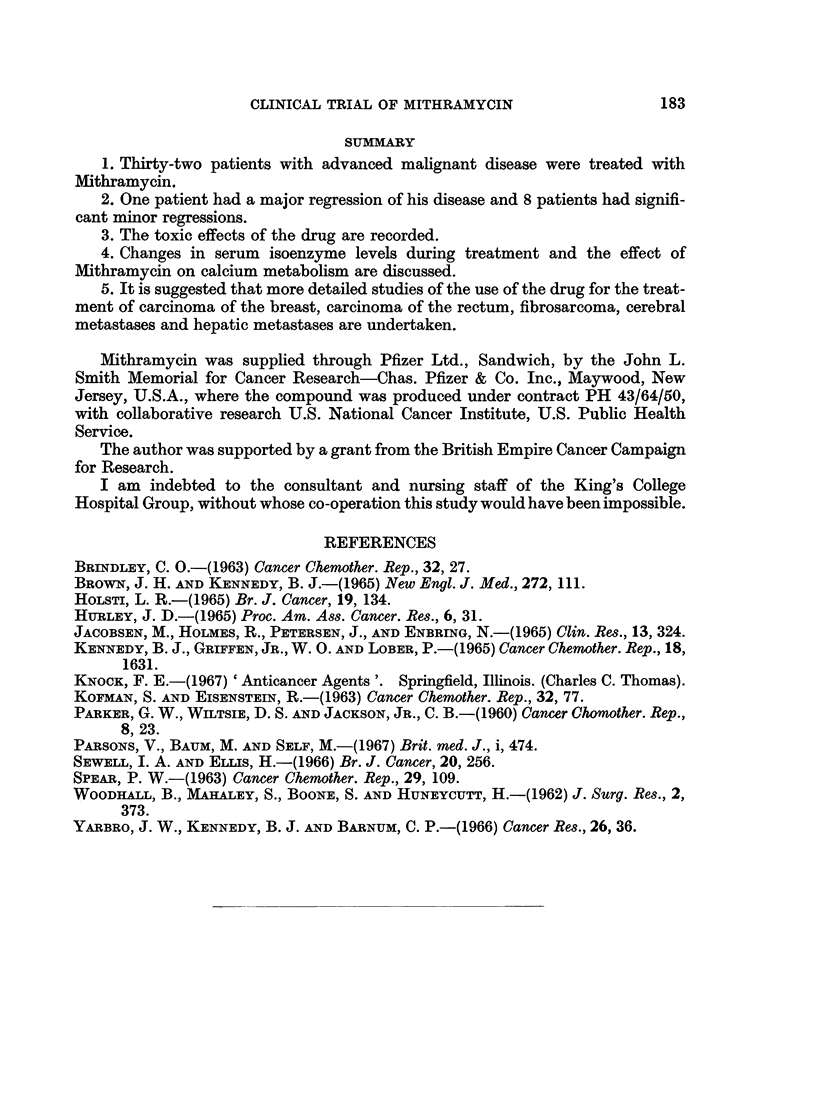# A clinical trial of mithramycin in the treatment of advanced malignant disease.

**DOI:** 10.1038/bjc.1968.25

**Published:** 1968-06

**Authors:** M. Baum


### A CLINICAL TRIAL OF MITHRAMYCIN IN THE TREATMENT OF ADVANCED MALIGNANT DISEASE

M. BAUM

*From the Surgical Unit, King's College Hospital Medical School, London*

Received for publication February 23, 1968

MITHRAMYCIN is an antibiotic with cytotoxic activity derived from an actinomycete of the Streptomyces genus. The drug acts like Actinomycin D by inhibiting DNA directed RNA synthesis (Yarbro, Kennedy and Barnum, 1966).

There have been several clinical trials of this drug reported by American workers (Parker, Wiltsie, and Jackson, 1960; Spear, 1963; Kofman and Eisenstein, 1963; Hurley, 1965), but so far only 1 trial reported from the United Kingdom (Sewell and Ellis, 1966).

Mithramycin appears to have a broad spectrum of activity but to date its importance has only been established in the treatment of testicular neoplasms (Kennedy, Griffen and Lober, 1965; Brown and Kennedy, 1965).

*Design of trial*

The present trial was conducted as a phase II investigation of a new anticancer agent according to the methodology of Brindley (1963). In phase I the toxic side effects of a new drug are established and a safe dosage schedule worked out. In phase II a large number of tumour types are screened for any evidence of antitumour activity however minimal. Then phase III trials are undertaken to follow up this evidence in a specific tumour type.

For a phase II trial the following criteria have to be met for each patient entered.

A. Incurable cancer which has failed to respond to previous conventional therapy.

B. Histological proof of diagnosis.

C. One or more of the following for the objective assessment of tumour regression.

- (i) Palpable tumour masses with well defined margins which can be measured by caliper or ruler.
- (ii) Multiple lesions that can be counted.
- (iii) Well defined bony or lung metastases that can be measured on an X-ray.
- (iv) A tumour-specific biochemical abnormality which can be measured quantitatively.

D. At least 1 month since prior treatment by cytotoxic drugs or radiotherapy.

E. No evidence of bone marrow depression or renal failure. No patients with, a white cell count of less than 3500/mm.$^3$, a platelet count of less than 100,000/mm.$^3$, or a blood urea of more than 40 mg./100 ml. were admitted to this particular trial.

*Therapeutic regime*

The dosage and method of administration was similar to that recommended by Brown and Kennedy (1965). The drug was kept at $-10°$ C. in vials containing 2·5 mg. of the crystalline material. 4·9 ml. of sterile water were used to dissolve the crystals, the resulting solution containing 0·5 mg. of Mithramycin/ml. 25 $\mu$g./kg. body weight of the solution were added to 500 ml. of 5 % dextrose and given slowly by continuous intravenous infusion over 12 hours. The drip was then kept open with another bottle of 5 % dextrose to complete the 24 hours before the next dose. If possible a 25 $\mu$g./kg. dose was given in each 24 hour period for 8 days. Sometimes the course was interrupted for mild toxic symptoms, or to give the patient " a rest " and occasionally the course had to be terminated because of the onset of severe toxic symptoms.

*Laboratory investigations*

Regular laboratory investigations were carried out before, during and for some days after the course of therapy according to Table I.

TABLE I.—*Laboratory Investigations*

| Time Interval | Investigation |
|---|---|
| Daily | Haemoglobin, total and differential white cell count and platelet count. Serum calcium and phosphorus. Urinary calcium, hydroxyproline and creatinine. |
| Alternate days (starting a week before and ending a week after treatment). | Serum lactic dehydrogenase (LDH), glutamic oxalic transaminase (SGOT), glutamic pyruvic transaminase (SGPT) and alkaline phosphatase (SAP) |
| Three day intervals | Blood urea and electrolytes. |

*Assessment of results*

The response to treatment was assessed by measuring or counting the tumours before and 2 days after the course of therapy. The grading of the results is shown in Table II.

TABLE II.—*Assessment of Response to Treatment*

| | |
|---|---|
| Major regression | 50% or more reduction in size or number of lesions. |
| Minor regression | Measureable reduction in size of tumour of less than 50% or reduction in number of lesions less than 50%. |
| No change | No change in size or number of lesions. |
| Progression | Increase in size or number of lesions. |

As some of the more interesting responses to the drug could not be classified adequately on this basis alone, special mention of individual cases will be made later in the paper. Duration of response was not recorded as this was not thought to be relevant to this phase of a trial.

Subjective responses were recorded and will be commented on in individual cases but no attempt to classify these on a numerical scale was made.

### RESULTS

Thirty-two patients were treated, 20 females and 12 males. The mean age of the group was 61 years and ranged from 42 years to 83 years. Eleven patients tolerated the full 8 day course and 1 patient had 2 full courses separated by 1 month. Fifteen patients had courses lasting 4 to 7 days and 6 patients had less than 4 days' treatment. The daily dose of Mithramycin ranged from 0·9 mg. to 2·0 mg. and the total dose of the drug ranged from 1·4 mg. to 14 mg. with a mean total dose of 8·8 mg.

The sites of the primary tumours and their histology, the number of days of treatment and the response to treatment, are listed in Table III.

TABLE III

| | Site of Primary | Histology | Number of Days of Treatment | Response |
|---|---|---|---|---|
| 1 | Bone Marrow | Myeloma | 8 | No change |
| 2 | Breast | Spheroidal cell carcinoma | 2 | Minor regression |
| 3 | ,, | Adenocarcinoma | 7 | Progression |
| 4 | ,, | ,, | 6 | Minor regression |
| 5 | Rectum | ,, | 8 | No change |
| 6 | Bronchus | Squamous cell carcinoma | 7 | Progression |
| 7 | Breast | Adenocarcinoma | 7 | Minor regression |
| 8 | Bronchus | Anaplastic carcinoma | 7 | Progression |
| 9 | ,, | Squamous cell carcinoma | 4 | No change |
| 10 | Rectum | Adenocarcinoma | 6 | Major regression |
| 11 | Breast | ,, | 8 | Minor regression |
| 12 | Uterus | ,, | 8 | ,, ,, |
| 13 | Breast | ,, | 7 | ,, ,, |
| 14 | ,, | ,, | 7 | No change |
| 15 | Rectum | ,, | 6 | Minor regression |
| 16 | Pancreas | ,, | 2 | No change |
| 17 | Rectum | ,, | 8 | Progression |
| 18 | ,, | ,, | 1 | ,, |
| 19 | Pancreas | ,, | 1 | ,, |
| 20 | Ovary | ,, | 8 | ,, |
| 21 | Breast | Spheroidal cell carcinoma | 2 | ,, |
| 22 | Unknown | Adenocarcinoma (hepatic metastases) | 8 | Minor regression |
| 23 | Breast | Adenocarcinoma | 3 | Progression |
| 24 | Pelvis | Fibrosarcoma | 8 × 2 | No change |
| 25 | Rectum | Adenocarcinoma | 8 | ,, ,, |
| 26 | Mouth | Squamous cell carcinoma | 8 | ,, ,, |
| 27 | Kidney | Adenocarcinoma | 7 | ,, ,, |
| 28 | Bronchus | Squamous cell carcinoma | 8 | Progression |
| 29 | Bronchus | Anaplastic carcinoma | 6 | No change |
| 30 | Pelvis | Fibrosarcoma | 8 | ,, ,, |
| 31 | Leg | ,, | 4 | Minor regression |
| 32 | Breast | Adenocarcinoma | 6 | No change |

There was 1 major regression occurring in a patient with carcinoma of the rectum. Nine minor regressions were recorded, 5 from patients with carcinoma of the breast and 1 each from patients with carcinoma of the rectum, carcinoma of the uterus, fibrosarcoma and hepatic secondaries. There was no apparent relation between length of treatment and response, indeed, most recorded regressions became obvious after only 2 or 3 days of therapy.

Ten patients died within 2 weeks of therapy, giving some indication of the advanced nature of the disease in a large proportion of the cases admitted to the trial.

Subjective improvement was commonly met with but difficult to interpret. A feeling of well-being could easily be accounted for on psychological grounds, resulting from the renewed interest shown in a patient who probably sensed that hope had been abandoned in their case. In addition to this, however, several patients who showed no objective signs of tumour regression noted marked improvement in their pain, leading to a reduced intake of analgesics. This could possibly be due to neurotoxicity as seen with other anticancer agents such as methotrexate (Woodhall, Mahaley, Boone and Huneycutt, 1962). We have been unable to confirm this yet with animal experiments.

*Illustrative case histories*

(1) *A.C.—male aged* 65.—He presented with a 3 month history of change in bowel habit. An abdomino-perineal resection was performed for carcinoma of the rectum. At operation the growth extended through the bowel wall. Six months later he returned complaining of perineal pain and urinary incontinence. There was a massive pelvic recurrence extending into the perineum as a superficial ulcerated lesion 8 cm. × 4 cm. He was given a course of Mithramycin but this had to be stopped after 6 days because of vomiting, although his platelet count remained above 100,000 cm. Following this the perineal growth became necrotic and his pain completely disappeared. Two days after finishing treatment he developed purpura and ecchymoses and started bleeding from the gums. A platelet count at this time was 16,000 cm. He was put on large doses of prednisone and within 5 days his platelet count had returned to within normal limits. However, he died a week later. Post mortem showed that the cause of death was bronchopneumonia but sections of the pelvic tumour showed extensive antemortem necrosis.

Comment: This case illustrates the principal danger of the drug, although the thrombocytopenia in this case did not occur while the drug was being given.

(2) *N.C.—female aged* 59.—A curettage to find the cause of post-menopausal bleeding confirmed the diagnosis of adenocarcinoma of the uterus. This was treated by a total hysterectomy and bilateral salpingo-oophorectomy from which she made a good recovery. Ten months later a pelvic recurrence was treated with radiotherapy. After a further 2 months she was admitted with right sided weakness, pains in the back, a severe headache and vomiting. A gamma scan of the brain using radioactive mercury confirmed the presence of cerebral metastases. She was given a full course of Mithramycin. After 2 days she developed cerebral oedema but this improved after a further 48 hours and by the end of the 8th day course, she was feeling well, free of pain, alert and active with a good appetite. There was some objective improvement in her C.N.S. signs as well. A constant fluid intake was maintained throughout so it was unlikely that the improvement resulted from inadvertent dehydration therapy. She maintained her subjective improvement for about 6 weeks and then slowly deteriorated and died a further month later.

Comment: Regression of cerebral metastases has been noted before with Mithramycin (Kofman and Eisenstein, 1963).

(3) *W.S.—male aged* 64.—A year before being referred for treatment he had a below knee amputation for a fibrosarcoma of low grade malignancy arising in the soft tissues around the ankle. Four months after this he developed lung metastases and was started on Methotrexate 5 mg. daily. Whilst on this therapy, metastatic deposits appeared in the amputation stump, in the groin, in the skin and in both humeri. Methotrexate was stopped and he was given repeated courses of radiotherapy without significant response.

In November, 1966 he was started on Mithramycin. After 3 doses the deposit in the amputation stump became necrotic and sloughed. After a further dose he developed massive ascites and bilateral pathological fractures of both humeri. Abdominal paracentesis revealed blood and necrotic debris. He died 3 days later. Post mortem examination showed massive peritoneal deposits all of which had undergone necrosis and both humeri were almost entirely replaced by necrotic tumour.

Comment: In this case the tumour was obviously highly sensitive to the drug but the sudden necrosis of such a large mass of malignant tissue contributed to his death.

(4) *A.S.—male aged* 63.—In 1961 he had an abdomino-perineal resection of the rectum performed for adenocarcinoma. In January, 1966 he was readmitted because of weight loss and perineal pain. On examination a hard fixed mass was noticed in the left iliac fossa extending 10 cm. above the anterior superior iliac spine and his liver extended 4 finger breadths below the costal margin. He was given a course of Mithramycin which was stopped after 6 days because of vomiting, weakness and dizziness. During treatment his pain disappeared completely and the mass in the left iliac fossa reduced in size. By the end of the course it extended 6 cm. above the iliac spine and 3 months later it had reduced to a mobile lump 3 cm. in diameter. He was able to return to work and has remained well until the time of writing—18 months later. However, the mass in his left iliac fossa has increased in size again and he has developed a metastasis in his buttock.

Comment: In retrospect this was the only patient in whom we achieved worthwhile regression. There has been a long-lasting subjective improvement as well as objective signs of tumour regression, at the expense of only mild and short-lived toxic side effects.

*Toxicity*

The incidence of toxic side effects is summarized in Table IV.

TABLE IV.—*Incidence of Toxic Side Effects*

| Toxic symptoms | No. of Patients |
|---|---|
| None | 10 |
| Anorexia and nausea | 9 |
| Severe vomiting | 5 |
| Thrombocytopenia | 4 |
| Diarrhoea | 3 |
| C.N.S. symptoms | 3 |
| Thrombophlebitis | 3 |

Toxic symptoms were relatively common and only 10 patients tolerated a full course without any unpleasant side effects. The commonest toxic symptoms were anorexia and nausea which were usually easy to control without the necessity of terminating the course of treatment. Uncontrollable vomiting was met with in 5 cases and was an absolute indication to stop therapy. Thrombocytopenia occurred in 4 patients and is probably the most dangerous side effect. In 2 patients the platelet count fell during therapy and rapidly returned to normal without ill effect on witholding the drug. In another 2 thrombocytopenia developed 2 days after the end of a course of Mithramycin and both developed purpura and bleeding. Steroid therapy was successful in correcting both. They emphasise the importance of daily platelet counts before, during and after therapy with Mithramycin.

Leucopenia never occurred but an unexplained polymorph leucocytosis was often seen. Severe diarrhoea troubled 3 patients and a further 3 developed C.N.S. symptoms such as drowsiness and dizziness.

The infusion caused a local thrombophlebitis in 3 patients and as this recurred after repeated repositionings of the infusion it was probably a true effect of the drug and not just due to faulty technique.

On no occasion was conclusive laboratory evidence of liver or kidney damage encountered.

*Changes in serum isoenzyme levels*

In order to test the drug for hepatotoxicity, liver function tests were carried out on patients before and during therapy. In particular SGOT, SGPT, LDH and SAP levels were carefully watched. An occasional patient developed a marked rise in LDH levels in the absence of a comparable rise in the levels of the other enzymes studied, and in the absence of other laboratory evidence of liver damage. Fractionation of the LDH into anaerobic and aerobic (heat labile and heat stable) components suggested that the major source of increase was in the anaerobic fraction, i.e. from an extrahepatic source. Similar findings have been noticed by other workers (Brown and Kennedy, 1965; Medrek, 1966, personal communication). Tumour cell necrosis probably causes the increase in serum LDH levels, which is suggested by the fact that all but one of the present series of patients who showed objective signs of tumour regression developed elevated LDH levels. The converse was also true. None of the patients without evidence of tumour regression developed similar changes in isoenzyme levels. The patient illustrated in Fig. 1 shows a typical pattern associated with tumour response. As the enzyme changes preceded the objective signs of regression, serial estimations of serum LDH might be used as evidence of tumour sensitivity to Mithramycin. Holsti (1965) has already suggested a similar method to assess the response of a tumour to radiotherapy.

*Effect on calcium metabolism*

The effect of Mithramycin on calcium metabolism was first noticed by Jacobsen, Holmes, Petersen and Enbring (1965). The changes in calcium metabolism during this trial were investigated fully and have already been reported (Parsons, Baum and Self, 1967).

Our findings suggested that Mithramycin blocked the peripheral action of parathyroid hormone or vitamin D on gut and bone leading to a consistent hypocalcaemic effect and a striking fall in urinary calcium excretion. We have treated a patient in whom disseminated malignant disease caused a life threatening hypercalcaemia of 17 mg./100 ml. After 48 hours therapy with Mithramycin it fell to 9·5 mg./100 ml. The serum calcium continued to fall to 5·5 mg./100 ml. but returned to normal on withholding the drug. Unfortunately the platelet count started to fall and further doses of Mithramycin were not given and the serum

FIG. 1.—Serum isoenzyme changes occurring in association with Mithramycin therapy—see case history (1).

LDH—Lactic dehydrogenase
SGOT—Serum glutamic oxalic transaminase
SGPT—Serum glutamic pyruvate transaminase calcium returned to supra normal levels. On this evidence it is suggested that Mithramycin may turn out to be a useful drug in the emergency treatment of hypercalcaemia.

### CONCLUSION

All cases referred to this trial were in very advanced stages of malignant disease having recurred after, or failed to respond to, conventional therapy. Because of this it would have been unrealistic to have expected any long term remissions. However, any evidence of tumour sensitivity is interesting and would suggest that a more intensive study of that specific tumour type would be worthwhile as a phase III trial. From our experience therefore it is suggested that Mithramycin should be further investigated for its effect on the following: carcinoma of the breast, carcinoma of the rectum, fibrosarcoma, cerebral metastases and hepatic metastases.

Unfortunately the drug has serious side effects and these were often seen in the very cases that seemed to show tumour sensitivity. In view of this it would perhaps be more ethical to await the development of a readily available method of testing a tumour sample for sensitivity against a battery of chemotherapeutic agents (Knock, 1967).

SUMMARY

1. Thirty-two patients with advanced malignant disease were treated with Mithramycin.

2. One patient had a major regression of his disease and 8 patients had significant minor regressions.

3. The toxic effects of the drug are recorded.

4. Changes in serum isoenzyme levels during treatment and the effect of Mithramycin on calcium metabolism are discussed.

5. It is suggested that more detailed studies of the use of the drug for the treatment of carcinoma of the breast, carcinoma of the rectum, fibrosarcoma, cerebral metastases and hepatic metastases are undertaken.

Mithramycin was supplied through Pfizer Ltd., Sandwich, by the John L. Smith Memorial for Cancer Research—Chas. Pfizer & Co. Inc., Maywood, New Jersey, U.S.A., where the compound was produced under contract PH 43/64/50, with collaborative research U.S. National Cancer Institute, U.S. Public Health Service.

The author was supported by a grant from the British Empire Cancer Campaign for Research.

I am indebted to the consultant and nursing staff of the King's College Hospital Group, without whose co-operation this study would have been impossible.

### REFERENCES

Brindley, C. O.—(1963) *Cancer Chemother. Rep.*, **32**, 27.

Brown, J. H. and Kennedy, B. J.—(1965) *New Engl. J. Med.*, **272**, 111.

Holsti, L. R.—(1965) *Br. J. Cancer*, **19**, 134.

Hurley, J. D.—(1965) *Proc. Am. Ass. Cancer. Res.*, **6**, 31.

Jacobsen, M., Holmes, R., Petersen, J., and Enbring, N.—(1965) *Clin. Res.*, **13**, 324.

Kennedy, B. J., Griffen, Jr., W. O. and Lober, P.—(1965) *Cancer Chemother. Rep.*, **18**, 1631.

Knock, F. E.—(1967) ' Anticancer Agents '. Springfield, Illinois. (Charles C. Thomas).

Kofman, S. and Eisenstein, R.—(1963) *Cancer Chemother. Rep.*, **32**, 77.

Parker, G. W., Wiltsie, D. S. and Jackson, Jr., C. B.—(1960) *Cancer Chomother. Rep.*, **8**, 23.

Parsons, V., Baum, M. and Self, M.—(1967) *Brit. med. J.*, i, 474.

Sewell, I. A. and Ellis, H.—(1966) *Br. J. Cancer*, **20**, 256.

Spear, P. W.—(1963) *Cancer Chemother. Rep.*, **29**, 109.

Woodhall, B., Mahaley, S., Boone, S. and Huneycutt, H.—(1962) *J. Surg. Res.*, **2**, 373.

Yarbro, J. W., Kennedy, B. J. and Barnum, C. P.—(1966) *Cancer Res.*, **26**, 36.